# Supplementary material for: Saccharomyces boulardii (CNCM I-745) improves intestinal damage in sepsis by remodeling intestinal flora structure
Source: Front Cell Infect Microbiol. 2025 Jul 31;15:1602792. doi: 10.3389/fcimb.2025.1602792 (PMC12350329; doi:10.3389/fcimb.2025.1602792)
Supplement: Supplementary file 1 [file DataSheet1.zip › summary/summary/7_Differential_analysis/2_LEfSe_result/Group_BW4_vs_BW0_0.05_3.0/LEfSe_Group_BW4_vs_BW0.cladogram.pdf]

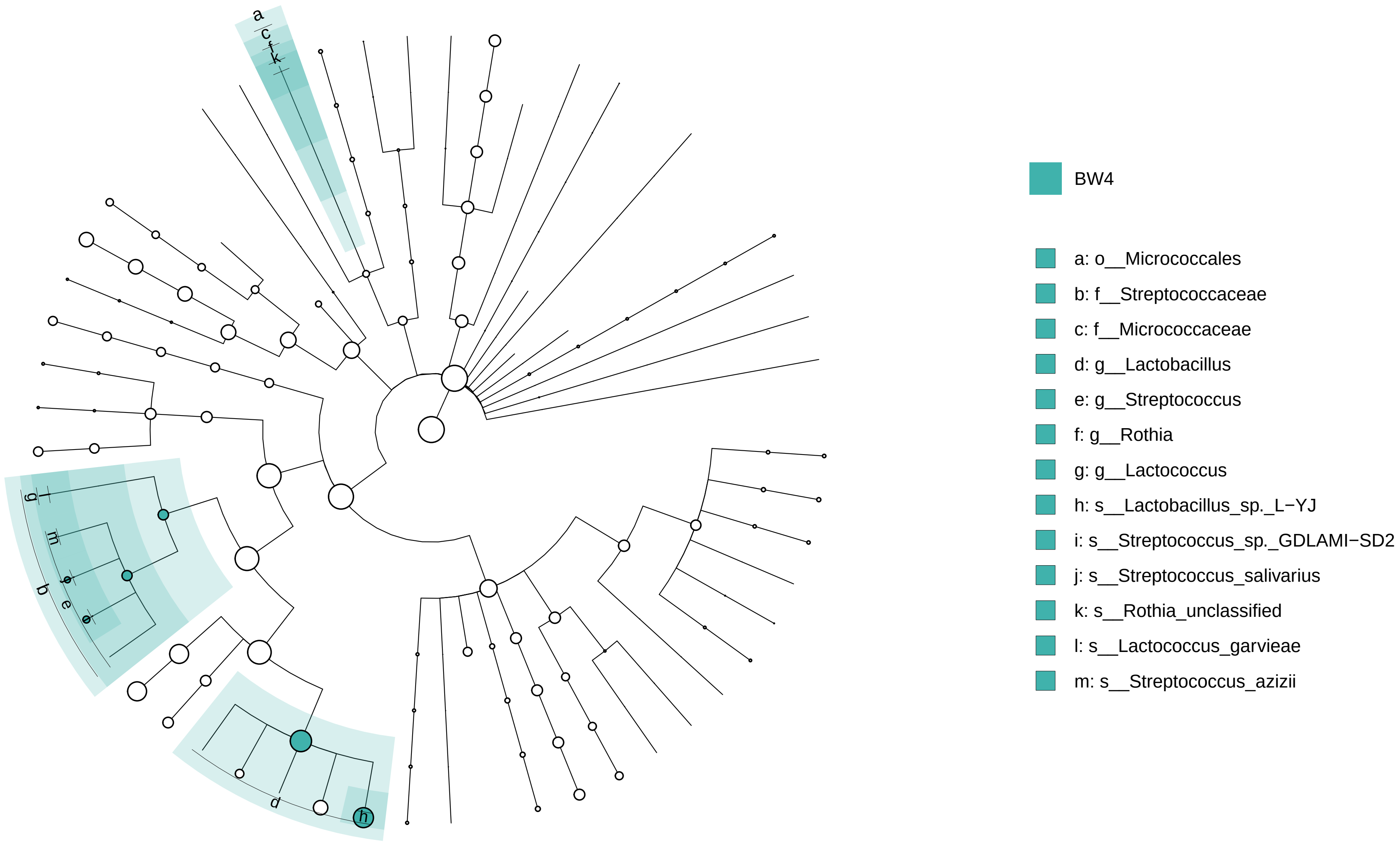

a
c
f
k
g
l
m
j
b
e
i
d
h
BW4
a: o__Micrococcales
b: f__Streptococcaceae
c: f__Micrococcaceae
d: g__Lactobacillus
e: g__Streptococcus
f: g__Rothia
g: g__Lactococcus
h: s__Lactobacillus_sp._L−YJ
i: s__Streptococcus_sp._GDLAMI−SD2
j: s__Streptococcus_salivarius
k: s__Rothia_unclassified
l: s__Lactococcus_garvieae
m: s__Streptococcus_azizii
